# Iodized Milk

**Published:** 1872-03

**Authors:** 


### Iodized Milk

From Hoffman's most admirable report on the "Progress of Pharmacy," we make the subjoined extract which has a practical value for the physician: It is well known that milk takes up iodine, disguising its taste, smell and color, completely: since iodine is an antiseptic, iodized milk keeps for some time. Dr. Hagar calls attention to this fact, and suggests that this, perhaps, is the mildest form of administering iodine. Its therapeutic effect seems to be equal only to about one-fifth of the iodine. Hagar thinks iodized milk will soon become a favorite form of administering iodine, and suggests the following mode of preparation: One part of iodine dissolved in ten parts of alcohol, admixed with ninety parts of fresh, warm cow's milk.—*Medical and Surgical Reporter*

